# Superior health-related physical fitness and well-being in 12–15-year-old Danish adolescents who are active in organized leisure-time sports – a cross-sectional study

**DOI:** 10.1371/journal.pone.0330950

**Published:** 2025-09-08

**Authors:** Christina Birch Meiner, Caroline Eckert, Charlotte Sandager Aggestrup, Kristina Pfeffer, Giampiero Tarantino, Camilla Prisak, Chiara Cimenti, Cecilie Thøgersen-Ntoumani, Nikos Ntoumanis, Peter Krustrup, Malte Nejst Larsen

**Affiliations:** 1 Department of Sports Science and Clinical Biomechanics, Sport and Health Sciences Cluster (SHSC), Faculty of Health Sciences, University of Southern Denmark, Odense, Denmark; 2 Danmarks Idrætsforbund (DIF), Brøndby, Denmark; 3 Danish Centre for Motivation and Behaviour Science (DRIVEN), University of Southern Denmark, Odense, Denmark; 4 Department of Sport, Exercise, and Rehabilitation Sciences, University of Birmingham, Birmingham, United Kingdom; 5 Danish Institute for Advanced Study (DIAS), University of Southern Denmark, Odense, Denmark; Instituto Politecnico de Santarem Escola Superior de Desporto de Rio Maior, PORTUGAL

## Abstract

**Background:**

Global reports indicate that less than 20% of 11–17-year-olds meet physical activity recommendations, and while organized sports participation increases the likelihood of meeting these guidelines, no other studies were found that examined the impact on well-being and physical fitness outcomes among Danish adolescents based on participation in leisure time sports.

**Methods:**

The study employed a cross-sectional design, assessing cardiorespiratory fitness, fat percentage, and well-being, as well as several other health and fitness outcomes among 1,333 Danish adolescents (50% girls). Differences between participants in organized sports and non-participants, as well as between participants in different sport categories were assessed through ANCOVA analysis.

**Results:**

Girls and boys participating in organized sports, ran longer (p<0.05) in the Yo-Yo Intermittent Recovery level1 Children's test (IR1C) (G: *d* = 0.72 [0.54; 0.89]; B: *d* = 0.70 [0.50; 0.89]) and had lower (p<0.05) fat percentages than non-participants (G: *d* = 0.42 [0.25; 0.59]; B: *d* = 0.33 [0.14; 0.51]). Organized sport participants had higher (*p < *0.05) well-being than non-participants in all KIDSCREEN-27 sub-scales apart from *social support and peers*. Girls in individual sports had lower (p<0.05) well-being than girls in other sports in all sub-scales apart from *parent relations and autonomy.* Multisport participation for both sexes, as well as participation in soccer for boys, was associated with a greater distance covered in the Yo-Yo IR1C (*p < *0.05). A significant drop out of sports from the previous to the current year was found among girls, but not among boys.

**Conclusion:**

Participation in organized leisure-time sports is linked to better health, fitness, and well-being, especially for girls, who show greater disparities with non-participants. Participation in team sports is linked to higher well-being compared to individual sports in girls, but not in boys. High dropout rates among adolescent girls call for targeted strategies to sustain their participation and derived benefits.

## Introduction

Physical activity (PA) is well established to have multiple beneficial and preventive effects on physical and mental health across the lifespan [1–3]. When zooming in on children and adolescents, research has shown positive associations between PA and both physical and psychological health indicators. For example, Biddle et al. [4], in their overview of reviews, found that PA may be beneficial for reducing depression levels among children and adolescents, while in relation to health status, research has shown that improved levels of PA result in better self-reported health [5] and objectively measured health indicators in school-aged children and youth [6]. Furthermore, it has been established that the effects of PA in childhood and adolescence not only affect the current health of individuals but extend well into adulthood. Research has also shown that high physical fitness and normal BMI in childhood and adolescence decrease the risk of accumulation of non-communicable disease risk factors, such as high blood pressure and high BMI [7–10] and significantly lower the risk of becoming overweight and obese in adulthood [11,12]. Huotari et al. [13] further showed that PA and fitness in adolescence significantly predicted PA levels in adulthood.

Despite the numerous health benefits of PA, children and adolescents (aged 5–17) do not move enough [14]. The World Health Organization (WHO) recommends 60 minutes of daily moderate-to-vigorous intensity physical activity (MVPA) for this population group [15]. However, the latest global reports show that more than 80% of children between 11–17-year-old do not meet the WHO recommendations [16]. The situation is similar in Denmark. A sub-study in the most recent Health-Behaviour in School-aged Children project (data collected in 2021/2022) showed that only one in five girls and one in four boys in 9^th^ grade accumulate 60 minutes of daily MVPA across a week [17].

A potential barrier to achieving the recommended PA levels is the type of PA in which children and adolescents engage. Several studies have shown that children and adolescents who participate in organized sports (club-based sports, fitness, after-school programs) in their leisure time have higher PA levels than their counterparts who only participate in self-organized sports (i.e., running in nature, playing soccer in the backyard or skateboarding with friends etc.) [18–21]. However, limited focus has been placed on the potential benefits of participation in organized sport on physical fitness and well-being. Prior research comparing health and fitness between adolescent participants and non-participants have focused mostly on mental health and ill-health, such as depression, anxiety and self-esteem measures [22–24], as well as physical measures relating to obesity status and cardiorespiratory fitness [24–27]. Results have predominantly shown better health scores for the organized sports participants. Significant differences between participants and non-participants have been reported among Danish children below the age of 12 years on a wide range of health, fitness and well-being variables. For example, among 8–10-year-olds, sports-participating children showed lower fat mass index (*d* = −0.37) and ran longer (*d* = 0.47) in the Yo-Yo Intermittent Recovery level 1 Children's test (IR1C) compared to non-participants [28]. Similar results have been shown within the 10–12 age group, with sports participating girls [29] and boys [30] performing better (F: *d* = 0.53, M: *d* = 0.69) in the Yo-Yo IR1C than their non-participating counterparts. In the 10–12 age group, the studies also found significant moderate differences in self-reported physical well-being between participants and non-participants, both among girls [29] (*d* = 0.50) and boys [30] (*d* = 0.60), with the organized sports participants having the higher scores. When looking at differences between sport types, soccer, in particular, has shown better beneficial effects on both well-being, health outcomes, fitness [29–31] and MVPA-levels [18].

In view of this evidence, there is a need to further investigate the differences in physical fitness and well-being between adolescents who participate in organized sports and those who do not. Such evidence could be used to make a stronger case for the role organized sports participation plays in keeping adolescents healthy, fit and thriving, and to support efforts to reduce adolescent drop out from organized sports [32]. Therefore, the objective of this paper is to investigate such differences. Additionally, as previous research suggests that girls in this age group are more likely to report experiencing feelings of sadness, anxiety, and self-doubt compared to boys [33], this paper will look into potential sex differences related to participation and discuss the role of sex when looking at the expected differences in physical performance [29,30] and well-being between sport participants and non-participants.

Thus, the primary aim of this paper was to explore differences in well-being and physical fitness outcomes among Danish adolescents aged 12–15 in relation to participation and non-participation in organized leisure time sports. The secondary aims of this research were to explore potential differences in the outcomes within participants of different sports, and to examine the magnitude of differences between sexes. Based on the above review of the mental and physical health benefits of physical activity [1–6], the association between organized sports participation and MVPA levels [18–21], and the results of similar studies in younger age groups [28–30], it is hypothesized that participation in organized leisure time sports would be associated with superior health, fitness, and well-being compared to non-participants. Differences are also expected between sexes and sport types. In this regard, it is hypothesized that girls will have higher BMI and fat percentages than boys, while boys will have better performance in physical fitness tests and better well-being. Furthermore, participants in team sports are expected to have higher well-being than participants in individual sports.

## Methods

### Study design and ethics statement

The study employed a cross-sectional design and used the baseline data from a large randomized controlled trial, the FIT FIRST Teen 10-week intervention study (ISRCTN Registry: ISRCTN76457599, registration date: 04/07/2024). This research was undertaken following the guidelines of the Declaration of Helsinki and an ethics waiver (S-20210099) was granted by the Health Research Ethics committee of the Southern Region of Denmark, waiving the obligation to report to the committee and informing the research team that written informed consent by study participants and their parents was not required for this type of study. The study was granted permission to collect and store personal data by the Research and Innovation Organization at the University of Southern Denmark (SDU RIO) (Opinion 11.695) following the guidelines for processing of personal data in research. Participants and their legal guardians received written information about the purpose, procedures and voluntary participation in the study, as well as the data retention period, legal basis for data processing and their rights to file a complaint with the Danish Data Protection Agency, through the schools’ internal communication platforms prior to the project start. This information was repeated (oral information) on the day of the testing.

### Setting

Recruitment of schools was undertaken in the spring of 2022 via posts on LinkedIn and advertisements in the Danish sports confederation’s newsletter. Private and public schools that were not special needs schools or dedicated sports schools were eligible for inclusion. Teachers wishing to participate with their classes contacted the project manager for further information and were included if eligible per the inclusion criteria. Testing was performed in August and September 2022, in the sports hall and surrounding rooms of each school, in the morning, as a part of the students’ normal school day, with at least one local teacher present during the testing procedures. Participants were not instructed to fast prior to testing and were permitted to drink water throughout the procedures. Given that the full testing session lasted approximately 90 minutes, which is comparable to a standard school lesson, food intake during the assessments was not permitted, except in cases where medical conditions necessitated it.

Prior to testing oral information was given and consent obtained. Participants were once again reminded that participation was completely voluntary and that they had the opportunity to withdraw their consent at any time without consequences. If a student did not consent to data collection, they were still allowed to participate in the physical testing without their results being recorded, in order to avoid feelings of exclusion based on participation status. Tests were performed by trained staff from the university in a standardized order starting with measurement of height and body composition, resting heart rate and blood pressure, and the stork single leg balance test. Hereafter, participants completed a shortened version of the FIFA 11+ for warm up [34], consisting of forward running, change of direction, jumping and plank holds. The testing continued with the standing long jump test and finally a collective Yo-Yo IR1C. Teachers were instructed to have the participants complete a self-administered online version of the KIDSCREEN-27 questionnaire [35] following the conclusion of the physical tests.

### Participants

In total, 1,333 participants between the ages of 12 and 15 years participated in the study. Students from 15 schools were included in the study, with participating classes from the 6^th^ grade (seven classes), 7^th^ grade (22 classes), 8^th^ grade (25 classes), and 9^th^ grade (17 classes). Both private and public schools were included, and all five Danish regions were represented, with participation from schools in both rural areas and large cities. A priori power calculations were not performed at the time the study commenced.

### Outcome measurements

#### Participation in organized leisure time sports.

During anthropometrical measurements participants were asked verbally about their participation in organized sports the previous and current year. A total of 54 different activities were identified and categorized into two main categories: ‘No organized sports (NOS)’ (No sports participation, scouts, self-organized sports) and ‘Organized sports participation (OSP)’. Participants in the OSP-group were further divided into four categories: ‘Soccer’, ‘Other team sports’ (handball, basketball, volleyball, other team sports), ‘Individual sports’ (gymnastics, badminton, tennis, horseback riding, dancing, gym activities, swimming, martial arts, boxing, padel, motocross, other individual sports) and ‘Multisport’ (participants active in multiple sports). The reason why soccer was categorized by its own is due to the hypothesis that participation in soccer would have the greatest impact on physical fitness and health status [29–31]. Further, the categorization in team sports and individual sports is based on the hypothesis that participating in team sports would provide better social interactions that can potentially lead to higher well-being compared to participating in individual sports [36–38]. A total of 135 participants were active in more than one sport and were included in the analysis as a separate multisport category.

#### Anthropometrics.

Height (cm) was measured using the Tanita, Leicester Height Measure Mk II, and body mass (kg), muscle mass (kg) and fat percentage (%) were measured using the InBody 270 bioimpedance analyzer, which has shown high accuracy and reliability for estimating body composition in children aged 10–12 years [39] (InBody270, Chungcheongnam-do 331–824, South Korea). Both measurements were undertaken with the participants barefoot and in light clothing. During data treatment, relative muscle mass (%) (muscle mass [kg]/total body mass [kg]*100) and BMI (body mass [kg]/height^2^[cm]) were calculated.

#### Blood pressure and resting heart rate.

Resting systolic (SBP, mmHg) and diastolic (DBP, mmHg) blood pressure and resting heart rate (RHR, bpm) were measured using an automated blood pressure monitor (M6 HEM-7322U-E; Omron, Hoofddorp, The Netherlands), which has been validated for accuracy in adults according to the ESH-IP2 protocol [40]. The cuff was placed on the participants left arm, directly on the skin, with the participants in the supine position. After an eight-minute rest period in a dark, quiet room, measures were taken three times with one minute in between recordings [41]. For statistical analyses the average of the two lowest recordings was used. Mean arterial pressure (MAP, mmHg) was calculated as 1/3 SBP + 2/3 DBP [41].

#### The stork single leg balance test.

Static balance was evaluated using the Stork test, which has shown moderate correlation with the Flamingo test, suggesting it may be suitable for measuring static balance in healthy individuals [42]. Participants were instructed to place their hands on their hips and their right foot on the medial side of the left knee. On the count of three-two-one-go the participant rose to their toes of the left foot and kept the position for as long as possible. When the position was broken, i.e., by moving the standing foot around, removing the foot from the knee or lifting the hands from the hips, time was stopped. Each participant had three tries on their left side followed by three tries on the right side. The tester was observing from the side, timing with a stopwatch and noting the time in seconds. The best of the six tries was used for analysis.

#### Paused standing long jump.

Jump length (cm) was measured with a paused, no-arms, standing long jump test. The standing long jump is strongly associated with lower body strength [43,44]. The participants were instructed to step to the start line, place their hands on their hips, squat down to a quarter squat and on the count of one-two-jump, do a long jump with a two-foot take-off and landing. The length of the jump was then measured from the starting line to the heel of the back foot. If the participants took their hands off their hips, did a single-foot take-off or failed the landing, the jump was not recorded, and an extra try was granted after being corrected. Two valid jumps were recorded for each participant, of which the best one was included in the analysis.

#### Yo-Yo intermittent recovery level 1 children’s test.

Cardiovascular fitness was measured with the Yo-Yo IR1C [45,46]. The Yo-Yo tests have been demonstrated to be valid and reliable tools for evaluating intermittent exercise capacity and aerobic performance in both elite and recreational young athletes [47]. The test was performed in a sports hall on a course with 16 meters running space and 4 meters recovery space behind the start/finish line. The participants were instructed to do repeated 2x16m shuttles at increasing speed until exhaustion, following the tempo of a recording for the original Yo-Yo Intermittent Recovery level 1 test. Between each 2x16 m shuttle, there was a 10 second recovery period, in which they were instructed to walk in the recovery space and get ready for the next shuttle. If the participants did not reach the line in time, ran short of the turning point, or had a false start, they received a warning. The test was over when the participants received a second warning or reached exhaustion. Teachers and scientific staff were placed at the start/finish line and at the turning point. When the participant finished the test, the level in which they ended the test was noted. The total distance was subsequently calculated from the level and used for analysis.

#### Questionnaire – KIDSCREEN-27.

A self-administered online version of the KIDSCREEN-27 questionnaire was used to measure well-being across five dimensions: physical well-being (PHY; 5 items), psychological well-being (PWB; 7 items), parents and autonomy (PAR; 7 items), social support and peers (SOC; 4 items) and school environment (SCH; 4 items) [35]. The KIDSCREEN-27 has shown good reliability and validity in European adolescent populations, with Cronbach’s alpha coefficients ranging from 0.79 to 0.84 across dimensions and has been validated for use in cross-cultural contexts [48]. Each item was accompanied by a 5-point Likert scale, with a higher score indicating higher well-being. Well-being scores were computed by transforming raw scores to t-scores in SPSS (IBM SPSS version 29.0.2.0 [20] (IBM SPSS Statistics, Chicago, IL)) using the official KIDSCREEN-27 SPSS-syntax provided on the KIDSCREEN website, prior to the data analysis in R studio. Thresholds for classifying KIDSCREEN test-values as, e.g., “normal” or “noticeable” was set to mean±0.5*SD of the scores for the European reference sample [49] per the KIDSCREEN manual. The questionnaire further contained questions regarding age, sex and participation in organized leisure time sports.

### Statistics

Statistical analyses were performed in R Studio (Version 2024.12.0 + 467, Posit Software, PBC). All data are presented as mean ± standard error (SE) unless stated otherwise and results with p-values <0.05 were considered statistically significant. The sample size varies for each outcome measure, as participation in tests was completely voluntary. Sample sizes for each test are reported in the tables. Data from girls and boys were analyzed separately and only participants reporting a specific sports category were included in subgroup analyses.

Differences in outcome variables across sex (girls, boys), sports club affiliation status (NOS, OSP) and sport categories (soccer, other team sports, individual sports, multisport) were investigated by ANCOVA using the *lm*-function from the *stats*-package in R studio [50]. For the analyses of physical outcomes, age and height were added as covariates in the model, while for the well-being outcomes, only age was included. The assumptions for the models were checked, and if the assumptions were violated, alternative approaches were used. For example, if normality of residuals was violated, variables were transformed (e.g., log transformation for positive-skewed variables) prior to the analysis [51]. Post hoc pairwise comparisons of differences between groups were made using the *emmeans* package in R studio [52]. A Tukey adjustment to control for multiple comparisons was used. Chi-Square tests were used to compare the proportion of sports participants between the previous year and the time of the testing. Cohen’s *d* was calculated for significant results and was interpreted using the following scale of magnitude: < 0.20 trivial; 0.20–0.49 small; 0.50–0.79 moderate; > 0.80: large [53].

## Results

### Descriptive statistics and sex differences

A total of 1,333 participants were included in the present analysis (Girls = 667, age (mean(Standard Deviation)): 13.7(1.0) years, height(mean(SD)): 163.3(9.1) cm; Boys = 666, age: 13.5(1.0) years, height: 169.5(9.8) cm). Table 1 shows the characteristics of the participants and the magnitude of the differences between girls and boys in the demographics and outcomes variables adjusted for age and height. Results revealed that boys had higher values in SBP and absolute and relative muscle mass (p < 0.001) than girls, whereas girls had higher values in DBP, RHR, body mass, BMI and fat percentage (p < 0.05). No differences were found in MAP. Regarding the physical fitness outcomes, analyses found that there were statistically significant large differences between the sexes in the Yo-Yo IR1C and standing long jump, and statistically significant small differences in the Stork balance stand, with boys outperforming girls (*p* < 0.001). Regarding well-being outcomes, findings showed that there were statistically-significant moderate differences in the PHY and PWB domains, as well as statistically significant small differences in the PAR, SOC and SCH domains, with boys reporting higher values (*p* < 0.001). Finally, the Chi-Square tests revealed no differences in the proportion of boys who reported being active and non-active in organized sport at the time of the study compared to the previous year: *X*^*2*^ (1, N = 583) = 0.104, p = 0.747. However, among the girls the test revealed statistically significant differences in the proportion who reported being active and non-active in organized sport at the time of the study compared to the previous year: *X*^*2*^ (1, N = 598) = 12.051, p < 0.001. There was a statistically significant difference between the proportions of OSP girls and boys at the time of the study, with a higher proportion of boys active than girls. All Cohen’s *d* values, and corresponding *p*-values can be found in Table 1.

**Table 1:** Differences between girls and boys in health, physical fitness and well-being.

	Girls	Boys	Cohens d [95% CI]	p-value
	**Physical outcome measures**			
**SBP (mmHg)**	102 (0.4) n = 652	105 (0.4) n = 648	0.29 [0.18; 0.40]	**<0.001**
**DBP (mmHg)**	63 (0.3) n = 652	61 (0.3) n = 648	0.26 [0.15; 0.37]	**<0.001**
**MAP (mmHg)**	76 (0.3) n = 652	76 (0.3) n = 648	0.00 [−0.11; 0.11]	0.267
**RHR (bpm)**	71 (0.4) n = 652	70 (0.4) n = 648	0.10 [−0.01; 0.21]	**0.034**
**Yo-Yo IR1C distance (m)**	528 (19.6) n = 630	886 (20.0) n = 608	0.73 [0.61; 0.84]	**<0.001**
**Standing long jump (cm)**	120.5 (0.9) n = 620	141.4 (0.9) n = 617	0.93 [0.82; 1.05]	**<0.001**
**Stork balance stand (sec)**	6.6 (0.3) n = 639	8.8 (0.3) n = 626	0.29 [0.18; 0.40]	**<0.001**
**Body mass (kg)**	58.9 (0.5) n = 643	57.0 (0.5) n = 647	0.15 [0.04; 0.26]	**0.005**
**BMI**	21.2 (0.2) n = 643	20.4 (0.2) n = 647	0.16 [0.05; 0.27]	**<0.001**
**Muscle mass (kg)**	23.7 (0.1) n = 638	25.6 (0.1) n = 645	0.75 [0.64; 0.86]	**<0.001**
**Muscle mass (%)**	40.7 (0.2) n = 638	45.3 (0.2) n = 645	0.91 [0.79; 1.02]	**<0.001**
**Fat percentage (%)**	25.3 (0.4) n = 638	17.7 (0.4) n = 645	0.75 [0.64; 0.86]	**<0.001**
**Sports participation previous year (%)**	68.1% n = 540	69.5% n = 554	*X*^*2*^ = 0.17	0.678
**Sports participation now (%)**	58.2% n = 598	68.6% n = 583	*X*^*2*^ = 14.54	**<0.001**
**Well-being**				
**Physical well-being**	45.0 (0.4) n = 511	51.3 (0.4) n = 501	0.66 [0.53; 0.78]	**<0.001**
**Psychological well-being**	43.3 (0.4) n = 511	49.5 (0.4) n = 499	0.66 [0.53; 0.78]	**<0.001**
**Parent relations and autonomy**	48.3 (0.5) n = 509	52.5 (0.5) n = 496	0.41 [0.29; 0.54]	**<0.001**
**Social support and peers**	48.4 (0.5) n = 509	50.6 (0.5) n = 495	0.21 [0.09; 0.33]	**0.001**
**School environment**	47.0 (0.4) n = 508	49.1 (0.4) n = 495	0.23 [0.11; 0.36]	**<0.001**

*SBP = systolic blood pressure, DBP = diastolic blood pressure, MAP = mean arterial pressure, RHR = resting heart rate, Yo-Yo IR1C=Yo-Yo intermittent recovery level 1 test modified for children, BMI = Body Mass Index, Mean (SE).*

### Differences between participants in organized sports and non-participants

Table 2 shows the characteristics and differences in the physical and well-being outcomes between students who participated in organized sports and those who did not. Statistically significant small-to-moderate differences (*d = *0.22–0.72, *p* < 0.05) were found between the groups on multiple outcomes, for both girls and boys. There was a statistically significant moderate difference in the Yo-Yo IR1C distance between OSPs and NOSs, both for girls and boys, with OSPs showing higher values (*p* < 0.001). There were small differences between the OSPs and the NOSs for both girls and boys on fat percentage, with OSP showing lower values (*p* < 0.001), and a moderate difference in the in relative muscle mass for girls, with the OSP showing higher values (*p* < 0.001). When comparing the KIDSCREEN27 well-being scores of the OSPs and the NOSs a statistically significant moderate difference was found in the PHY domain among girls and a small difference among boys with OSP showing higher values (*p* < 0.001). Statistically significant small differences were also found between OSPs and NOSs in girls in both the PWB and PAR domains, as well as in both sexes in the SCH domain, with the OSPs reporting higher well-being across the four sub-scales (*p* < 0.05). No differences were found between OSPs and NOSs for either sex in the SOC domain. All Cohen’s *d* values, and corresponding *p*-values can be found in Table 2.

**Table 2:** Health, physical fitness and well-being as a function of participation in organized leisure-time sports.

	Girls						Boys				
	NOS	OSP	Cohens *d* [95% CI]		p-value			NOS	OSP	Cohens* d* [95% CI]	p-value
**Physical outcome measures**											
**SBP (mmHg)**	101 (0.5) n = 232	101 (0.4) n = 325		0.00 [−0.17; 0.17]		0.502		106 (0.8) n = 164	106 (0.5) n = 375	0.00 [−0.18; 0.18]	0.536
**DBP (mmHg)**	64 (0.4) n = 232	62 (0.4) n = 325		0.27 [0.10; 0.44]		**<0.001**		63 (0.6) n = 164	60 (0.4) n = 375	0.39 [0.20; 0.57]	**0.003**
**MAP (mmHg)**	77 (0.4) n = 232	75 (0.3) n = 325		0.31 [0.14; 0.48]		**0.002**		77 (0.6) n = 164	75 (0.4) n = 375	0.26 [0.07; 0.44]	**0.005**
**RHR (bpm)**	75 (0.7) n = 232	70 (0.6) n = 325		0.47 [0.29; 0.64]		**<0.001**		72 (0.9) n = 164	68 (0.6) n = 375	0.35 [0.16; 0.53]	**<0.001**
**Yo-Yo IR1C distance (m)**	387 (19.5) n = 225	598 (16.8) n = 310		0.72 [0.54; 0.89]		**<0.001**		629 (46) n = 151	1024 (30) n = 355	0.70 [0.50; 0.89]	**<0.001**
**Standing long jump (cm)**	112.8 (1.3) n = 226	122.2 (1.1) n = 300		0.49 [0.31; 0.66]		**<0.001**		138.0 (1.8) n = 151	146.2 (1.2) n = 363	0.36 [0.17; 0.55]	**<0.001**
**Stork balance stand (sec)**	6.2 (0.4) n = 230	7.2 (0.4) n = 316		0.15 [−0.02; 0.32]		0.068		6.9 (0.7) n = 161	9.3 (0.5) n = 356	0.26 [0.07; 0.45]	**0.003**
**Body mass (kg)**	57.4 (0.7) n = 229	55.5 (0.6) n = 321		0.18 [0.01; 0.35]		**0.035**		60.9 (0.9) n = 167	59.1 (0.6) n = 371	0.16 [−0.03; 0.34]	0.113
**BMI**	21.4 (0.3) n = 229	20.7 (0.2) n = 321		0.18 [0.00; 0.34]		**0.036**		21.0 (0.3) n = 167	20.4 (0.2) n = 371	0.16 [−0.03; 0.34]	0.083
**Muscle mass (kg)**	22.0 (0.2) n = 226	22.4 (0.1) n = 319		0.17 [0.00; 0.34]		**0.026**		26.7 (0.2) n = 166	27.2 (0.2) n = 371	0.14 [−0.04; 0.33]	0.067
**Muscle mass (%)**	39.0 (0.3) n = 226	41.0 (0.2) n = 319		0.50 [0.33; 0.68]		**<0.001**		44.7 (0.5) n = 166	46.4 (0.3) n = 371	0.28 [0.10; 0.47]	**0.003**
**Fat percentage (%)**	27.7 (0.5) n = 226	24.6 (0.4) n = 319		0.42 [0.25; 0.59]		**<0.001**		19.2 (0.7) n = 166	16.1 (0.5) n = 371	0.33 [0.14; 0.51]	**<0.001**
**Well-being**											
**Physical well-being**	42.2 (0.6) n = 237	47.5 (0.6) n = 262		0.56 [0.38; 0.74]		**<0.001**		48.2 (0.7) n = 178	53.0 (0.5) n = 318	0.49 [0.31; 0.68]	**<0.001**
**Psychological well-being**	42.2 (0.6) n = 237	44.3 (0.6) n = 262		0.22 [0.05; 0.40]		**0.009**		48.3 (0.8) n = 178	50.2 (0.6) n = 316	0.18 [0.00; 0.36]	**0.044**
**Parent relations and autonomy**	46.9 (0.7) n = 235	49.5 (0.6) n = 262		0.25 [0.08; 0.43]		**0.005**		51.3 (0.8) n = 177	53.3 (0.6) n = 314	0.19 [0.00; 0.37]	**0.037**
**Social support and peers**	47.9 (0.7) n = 235	48.8 (0.7) n = 262		0.08 [−0.9; 0.26]		0.363		50.0 (0.8) n = 176	51.0 (0.6) n = 314	0.09 [−0.09; 0.28]	0.367
**School environment**	45.8 (0.6) n = 235	48.0 (0.5) n = 261		0.26 [0.08; 0.43]		**0.005**		47.6 (0.7) n = 176	50.0 (0.5) n = 314	0.27 [0.08; 0.45]	**0.010**

*NOS = No Organized Sports, OSP = Organized Sports Participant. SBP = systolic blood pressure, DBP = diastolic blood pressure, MAP = mean arterial pressure, RHR = resting heart rate, Yo-Yo IR1C=Yo-Yo intermittent recovery level 1 test modified for children, BMI = Body Mass Index, Mean (SE).*

### Differences between participants in different sport categories

Tables 3 and 4 show the results of the ANCOVA and post-hoc comparisons between the four sport categories *‘soccer’ (SC)*, *‘other team sports’ (OTS)*, *‘individual sports’ (IS)* and *‘multisport’ (MS)* within the OSPs for girls and boys respectively. Participants reporting participation in organized sports who did not specify which sport they participated in were not included in the present analyses (n = 18). The ANCOVA showed significant differences between the sport categories on multiple outcomes for both girls and boys. Post-hoc pairwise comparisons revealed that girls participating in multiple sports and a team sport other than soccer, ran significantly longer than participants in individual sports (MS: *d* = 0.61: 95% CI: 0.28 to 0.94, *p* = 0.002; OTS: *d* = 0.41: 95% CI: 0.10 to 0.71, *p* = 0.015). Furthermore, girls in other team sports had significantly higher absolute muscle mass than girls in individual sports (*d* = 0.40: 95% CI: 0.10 to 0.71, *p* = 0.029). Among boys, the post-hoc comparisons revealed that boys who participated in soccer ran longer in the Yo-Yo IR1C test compared to individual sports participants (*d* = 0.74: 95% CI: 0.47 to 1.01, *p* < 0.001), and other team sports players (*d* = 0.69: 95% CI: 0.29 to 1.09, *p* = 0.024). Multisport participants also ran significantly longer than individual sports participants (*d* = 0.40: 95% CI: 0.19 to 0.75, *p* = 0.011). Furthermore, the post hoc comparisons showed that male multisport participants had higher body mass, BMI and absolute muscle mass than soccer players (body mass: *d* = 0.42: 95% CI: 0.12 to 0.71, *p* = 0.021; BMI: *d* = 0.41: 95% CI: 0.12 to 0.71, *p* = 0.019; muscle mass: *d* = 0.41: 95% CI: 0.12 to 0.71, *p* = 0.015). Soccer players further had significantly lower fat percentage than individual sports participants (*d* = 0.36: 95% CI: 0.11 to 0.62, *p* = 0.041).

**Table 3:** Girls’ health, physical fitness and KIDSCREEN27 well-being scores in relation to specific leisure-time sports categories.

	Soccer	Other team sports	Individual sports	Multisport	F	p-value
**Physical outcomes**						
**SBP (mmHg)**	100 (1.1) n = 48	101 (1.1) n = 58	101 (0.6) n = 165	100 (1.2) n = 46	4.99	**<0.001**
**DBP (mmHg)**	62 (0.9) n = 48	61 (0.9) n = 58	66 (0.5) n = 165	63 (1.0) n = 46	1.01	0.415
**MAP (mmHg)**	75 (0.9) n = 48	74 (0.8) n = 58	75 (0.5) n = 165	76 (0.9) n = 46	1.71	0.133
**RHR (bpm)**	70 (1.5) n = 48	69 (1.4) n = 58	70 (0.8) n = 165	69 (1.5) n = 46	1.79	0.115
**Yo-Yo IR1C distance (m)**	581 (53.7) n = 43	683 (48.5) c n = 58	541 (27.3) bd n = 158	752 (52.0) c n = 47	6.20	**<0.001**
**Standing long jump (cm)**	121.5 (3.0) n = 42	123.5 (2.7) n = 57	122.0 (1.5) n = 153	124.1 (2.9) n = 44	3.16	**0.009**
**Stork balance stand (sec)**	6.3 (1.0) n = 47	8.0 (0.9) n = 59	7.1 (0.5) n = 158	7.0 (1.0) n = 47	3.02	**0.011**
**Body mass (kg)**	57.0 (1.3) n = 48	56.7 (1.3) n = 55	55.3 (0.7) n = 165	54.2 (1.4) n = 44	21.79	**<0.001**
**BMI**	21.2 (0.5) n = 48	21.0 (0.5) n = 55	20.5 (0.3) n = 165	20.1 (0.5) n = 44	1.96	0.085
**Muscle mass (kg)**	22.8 (0.3) n = 48	23.3 (0.3) c n = 55	22.3 (0.2) b n = 164	22.8 (0.3) n = 44	85.33	**<0.001**
**Muscle mass (%)**	40.5 (0.6) n = 48	41.5 (0.5) n = 55	40.9 (0.3) n = 164	42.3 (0.6) n = 44	4.03	**0.001**
**Fat percentage (%)**	25.6 (1.1) n = 48	24.3 (1.0) n = 55	24.6 (0.6) n = 164	22.3 (1.1) n = 44	2.71	**0.021**
**Well-being**						
**Physical well-being**	48.6 (1.2) n = 53	49.4 (1.3) c n = 48	44.9 (0.8) bd n = 124	52.2 (1.5) c n = 37	6.19	**<0.001**
**Psychological well-being**	46.5 (1.2) c n = 53	45.5 (1.2) n = 48	42.2 (0.8) ad n = 124	46.6 (1.4) c n = 37	4.10	**0.003**
**Parent relations and autonomy**	51.6 (1.5) n = 53	50.4 (1.6) n = 48	47.6 (0.9) n = 124	52.2 (1.7) n = 37	3.43	**0.009**
**Social support and peers**	51.8 (1.4) c n = 53	51.3 (1.5) c n = 48	46.5 (0.9) ab n = 124	49.3 (1.7) n = 37	3.95	**0.004**
**School environment**	46.9 (1.2) d n = 52	49.6 (1.2) n = 48	46.8 (0.8) d n = 124	52.1 (1.4) ac n = 37	4.77	**0.001**

*SBP = systolic blood pressure, DBP = diastolic blood pressure, MAP = mean arterial pressure, RHR = resting heart rate, Yo-Yo IR1C=Yo-Yo intermittent recovery level 1 test modified for children, BMI = Body Mass Index, F = F statistic value. Mean (SE). Physical outcomes adjusted for age and height. Well-being adjusted for age. a: significantly different from soccer, b: significantly different from other team sports, c: significantly different from individual sports, d: significantly different from multisport.*

**Table 4:** Boys’ health, physical fitness and KIDSCREEN27 well-being scores in relation to specific leisure-time sports categories.

	Soccer	Other team sports	Individual sports	Multisport	F	p-value
**Physical outcomes**						
**SBP (mmHg)**	105 (1.0) n = 104	108 (1.8) n = 34	106 (0.8) n = 148	104 (1.2) n = 79	12.90	**<0.001**
**DBP (mmHg)**	61 (0.7) n = 104	62 (1.3) n = 34	61 (0.6) n = 148	59 (0.8) n = 79	0.86	0.511
**MAP (mmHg)**	76 (0.7) n = 104	77 (1.2) n = 34	76 (0.6) n = 148	74 (0.8) n = 79	2.75	**0.019**
**RHR (bpm)**	67 (1.1) n = 104	72 (1.9) n = 34	70 (0.9) n = 148	66 (1.3) n = 79	5.14	**<0.001**
**Yo-Yo IR1C distance (m)**	1289 (61.7) bc n = 95	873 (102.0) a n = 35	843 (51.8) ad n = 137	1128 (69.2) c n = 78	9.28	**<0.001**
**Standing long jump (cm)**	146.8 (2.3) n = 99	143.9 (3.9) n = 35	145.1 (1.9) n = 138	147.3 (2.6) n = 81	9.81	**<0.001**
**Stork balance stand (sec)**	8.3 (1.0) n = 94	8.9 (1.7) n = 33	10.4 (0.8) n = 141	8.6 (1.1) n = 79	2.44	**0.034**
**Body mass (kg)**	56.9 (1.1) d n = 104	60.0 (1.9) n = 34	59.8 (0.9) n = 142	61.5 (1.2) a n = 81	47.47	**<0.001**
**BMI**	19.6 (0.4) d n = 104	20.6 (0.6) n = 34	20.6 (0.3) n = 142	21.2 (0.4) a n = 81	5.53	**<0.001**
**Muscle mass (kg)**	26.9 (0.3) d n = 104	27.3 (0.5) n = 34	27.2 (0.2) n = 142	28.1 (0.3) a n = 81	265.40	**<0.001**
**Muscle mass (%)**	47.4 (0.6) n = 104	45.9 (1.0) n = 34	45.9 (0.5) n = 142	46.1 (0.7) n = 81	11.90	**<0.001**
**Fat percentage (%)**	14.1 (0.8) c n = 104	16.8 (1.5) n = 34	17.1 (0.7) a n = 142	16.8 (0.9) n = 81	7.69	**<0.001**
**Well-being**						
**Physical well-being**	54.5 (0.8) c n = 137	52.9 (1.4) n = 45	51.1 (1.0) a n = 100	53.2 (1.6) n = 37	1.89	0.112
**Psychological well-being**	51.4 (0.9) n = 137	50.4 (1.6) n = 43	49.6 (1.0) n = 100	47.8 (1.7) n = 37	1.12	0.346
**Parent relations and autonomy**	54.1 (0.9) n = 136	53.0 (1.6) n = 42	52.5 (1.0) n = 100	52.7 (1.1) n = 37	0.42	0.792
**Social support and peers**	52.4 (0.9) n = 136	49.7 (1.7) n = 42	50.8 (1.1) n = 100	48.4 (1.8) n = 37	1.91	0.108
**School environment**	50.1 (0.8) n = 136	50.5 (1.4) n = 42	50.8 (0.9) n = 100	46.9 (1.5) n = 37	1.36	0.246

*SBP = systolic blood pressure, DBP = diastolic blood pressure, MAP = mean arterial pressure, RHR = resting heart rate, Yo-Yo IR1C=Yo-Yo intermittent recovery level 1 test modified for children, BMI = Body Mass Index, F = F statistic value. Mean (SE). Physical outcomes adjusted for age and height. Well-being adjusted for age. a: significantly different from soccer, b: significantly different from other team sports, c: significantly different from individual sports, d: significantly different from multisport.*

When comparing the well-being scores between sport categories within the OSPs among girls, the ANCOVA revealed statistically significant differences in all five subscales. Post-hoc comparisons revealed that individual sports participants had lower scores in the PHY domain than those in multiple sports (*d* = 0.81: 95% CI: 0.44 to 1.19, *p* < 0.001) and other team sports (*d* = 0.50: 95% CI: 0.17 to 0.84, *p* = 0.018). Participants in individual sports also had the lowest scores on the PWB domain, which were significantly lower than soccer players (*d* = 0.49: 95% CI: 0.16 to 0.81, *p* = 0.013) and multisport participants (*d* = 0.50: 95% CI: 0.13 to 0.87, *p* = 0.033). In the SOC domain, the individual sport participants scored significantly lower than those in soccer (*d* = 0.53: 95% CI: 0.20 to 0.85, *p* = 0.011) and other team sports (*d* = 0.47: 95% CI: 0.14 to 0.81, *p* = 0.032). Finally, the multisport participants had significantly higher scores in the SCH domain than those in soccer (*d* = 0.61: 95% CI: 0.17 to 1.04, *p* = 0.025) and individual sports (*d* = 0.60: 95% CI: 0.23 to 0.97, *p* = 0.005). No pairwise differences were found in the PAR domain.

Among boys, the ANCOVA did not reveal any statistically significant differences. However, the pairwise comparison showed that boys in soccer had significantly higher scores in the PHY domain than boys in individual sports (*d* = 0.35: 95% CI: 0.09 to 0.61, *p* = 0.035).

## Discussion

This study aimed to investigate differences in health-related physical fitness and well-being among 12–15-year-old Danish adolescents in relation to participation in organized leisure time sports. The main findings were that both girls and boys who engaged in organized leisure-time sports exhibited superior endurance capacity, had higher muscle mass and lower fat percentage, and reported higher well-being compared to their non-participating peers.

As expected, results from this study revealed that boys had better performance than girls in physical fitness outcomes. These results are consistent with previous research that has showed small to large differences in such outcomes between sexes [54]. Such results are not surprising and reflect the biological differences between the sexes, and the lower sport participation and PA levels among girls at this age. Further, these results are also aligned with the findings presented in this study, that girls tend to drop sport earlier than boys. Another expected finding showed that sport participation has a positive association with physical fitness, which has also been reported in studies conducted in other countries [27,55].

Results from this study differ from those reported in other papers when considering differences between sport categories. A Finish study investigating the differences in physical fitness performance between single sport participants and multisport among participants aged 12 years old found that boys participating in multiple sports performed significantly better than single sport participants in aerobic capacity and strength, (20-m shuttle run test, five leap jump test and push up test) [56]. The present study did not find such differences, except in aerobic capacity when comparing multisport to individual sports only. Further, this study showed that soccer is a stronger predictor of Yo-Yo IR1C test performance for boys than other types of sports. These results are aligned with those found in Portuguese adolescents as well as younger Danish children [45,46,57]. A potential explanation can be found in the nature of the game, consisting of great volumes of running at different speeds, covering long distances, as well as many changes of direction leading to a high aerobic as well as anaerobic training load [58–60].

The study also showed significant differences in body composition outcomes between boys and girls. This is consistent with previous studies, and it was expected considering the sex specific differences in storage of fat tissue [61–63]. However, the study also found that both girls and boys participating in organized sports had lower fat percentage than their non-participating counterparts. These results are consistent with results of a recent systematic review and meta-analysis by Mateo-Orcajada et al. [64], which explored the differences of physical fitness and body composition outcomes between active and sedentary adolescents aged 12–16. Mateo-Orcajada et al. [64] found that active adolescents had significantly lower fat percentages than their sedentary peers. These patterns have also been found in a longitudinal study examining the influence of sports participation on PA, fitness and body fat [25]. Once again, male soccer players showed significantly lower fat percentages compared to individual sports participants, and significantly lower BMI than boys participating in multiple sports.

A possible explanation for the superior fitness levels and body composition found in organized sports participants compared to non-participants is the elevated PA that has been consistently associated with leisure-time sports participation [18–21]. In a study by Kokko et al. [19], European adolescents were found to be 2.4–6.4 times more likely to meet the recommendations for weekly overall PA and 2.8–5.0 more likely to meet the vigorous intensity PA recommendations by participating in organized sports, compared to non-participants. Nielsen et al. [37] have further suggested the high transferability of soccer to school recess settings as a possible explanation for higher MVPA-levels in soccer players compared to participants in different sports, as well as non-participants, that could ultimately explain the higher fitness levels, higher muscle mass and lower fat percentages.

The present study showed statistically significant differences in both DBP and MAP results with OSPs presenting with lower values than NSPs among both girls and boys. However, although the differences are statistically significant, they are unlikely to be clinically meaningful as day-to-day and even hour-to-hour differences exceed the range of differences found in the present study (1–3 mmHg) [65].

Well-being scores for both girls and boys fall within the normal range of the European adolescent reference population [49]. The present study confirmed that there are differences in well-being between the sexes, with boys scoring significantly higher in all the well-being domains. This is in agreement with the findings of Kjellenberg et al. [66], who found significantly higher well-being scores in boys than girls using the KIDSCREEN-10 questionnaire. Such differences can be partly attributed to hormonal fluctuations during puberty, such as changes in estrogen and progesterone levels, which can increase emotional reactivity [33]. Additionally, societal pressures surrounding appearance and relationships are often more intense for girls and contribute to emotional distress [67].

Results from the present study showed that both sexes have better well-being (except *social support and peers)* if they are active in organized sports, compared to non-participants*,* with non-participating girls scoring noticeably low on *physical* and *psychological well-being,* indicating feeling *“physically exhausted”, “low energy”, “unfit”* and *“feeling depressed and unhappy”* [48]. Team-based sports seemed to have a strong positive effect with significantly higher values within *physical well-being* for both sexes as well as *psychological well-being* and *social support and peers* for girls when comparing with participants in individual sports. This is in line with similar studies conducted in 10–12-year-old children and may be related to the sense of achievement and goal-setting inherent in sports that can boost self-esteem and resilience [68]. Adolescents involved in team sports may also benefit from a sense of belonging and identity within their team, contributing to overall happiness and life satisfaction [69]. Further, participating in multiple sports was associated with significantly higher scores in the *school environment* subscale for girls compared to soccer and individual sports. Research has extensively suggested that participation in multiple extracurricular activities, including sports, is positively associated with academic achievement [70], and stronger connections to their school [71], which positively influences students’ academic motivation and well-being. This may lead to a better perception of the school environment, including a better sense of happiness within the school context, and stronger good relationships with teachers among adolescent girls.

The study also showed a significant drop out of organized sports among the girls. This is consistent with results from previous studies and reports that have widely shown higher rates of sports dropout among adolescent girls [72,73]. This is highly concerning when relating the results of the present study to results of similar studies in conducted with younger Danish children, since it shows that the magnitude of the differences in both physical fitness and well-being outcomes between participating and non-participating girls increases with age. For example, when comparing results from a study conducted by Madsen et al. [29] on differences in well-being between participating and non-participating girls aged 10–12 to the results of our study (12–15 year olds), there is an increase in the magnitude of the differences in the *physical well-being* (10–12yo: *d*: 0.5 vs 12–15yo: *d*: 0.6), *psychological well-being* (10–12yo: *d*: 0.1 vs 12–15yo: *d*: 0.2) and *school environment* (10–12yo: *d*: 0.2 vs 12–15yo: *d*: 0.3) sub-scales. Further, when looking at Yo-Yo IR1C performance in girls, Larsen et al. [28] found a small significant difference (*d*: 0.25) between active and non-active 8–10-year-olds, while Madsen et al. [29] found a moderate significant difference (*d*: 0.53) when investigating the differences among 10–12-year-olds, and the present study found an even larger difference in the Yo-Yo IR1C performance (*d*: 0.72). Thus, it seems we are left with a double problem: as the girls get older, we see a growing decrease in participation with a concurrent increase in differences in both physical and mental outcomes between female participants and non-participants.

Contrary to what was seen among the girls, no significant drop out of organized sports was seen among boys. When comparing differences in physical fitness and well-being outcomes between male participants and non-participants in the present study to those in studies of younger Danish boys, the differences in both well-being and physical outcomes are aligned [30].

This study is the first study to investigate such outcomes and differences in this age group in Denmark and will contribute to the literature in terms of understanding how participation in organized sport helps children and adolescents be more active and fit. Further, it will be a valuable addition to the body of research already existing for younger Danish populations, helping to understand how the differences between participants and non-participants vary with age. The results of this study, along with the growing body of research showing similar findings in other age groups and nationalities, highlight the importance of participation in organized sports. This study underscores a need for action to maintain the high participation rates reported among children, throughout adolescence, with a special focus on adolescent girls. A specific practical step ought to focus on educating coaches in organized sports to motivate children and pay special attention to the needs and well-being of adolescent girls, to retain their participation in organized sports. The results of this study provide important knowledge for policy makers, parents and other key figures influencing the lives of adolescents, and should be considered carefully, in discussion and decision-making processes in the field.

This study has several strengths, including the collection of a wide variety of data on both well-being and fitness and health outcomes, as well as the inclusion of participants from across the country and from different schools in relation to size, structure and environmental settings (rural/urban), which enhances the generalizability of the findings. However, there are also limitations to consider. The recruitment of schools on a voluntary basis increases the risk of participation bias, potentially affecting the representativeness of the sample. Additionally, the cross-sectional design of the study restricts the ability to definitively conclude that organized sports participation causes the observed differences in well-being and physical outcomes. It should also be noted that grouping diverse sports into broad categories may mask sport specific differences, meaning that differences in outcomes could differ if analyzed separately. However, due to the small numbers of participants in each sport, this was not feasible. Lastly, due to the absence of PA level measurements, it is not possible to examine the extent to which PA mediates the relationship between sports participation and the observed benefits. These limitations highlight the need for future research to employ longitudinal designs, that combines objective measurements of PA with information about sports participation, and evaluations of fitness and well-being outcomes to better elucidate these relationships. Furthermore, future research should focus on identifying patterns in, and underlying causes of, drop out of organized sports among adolescents, as well as proposing possible solutions to the problem.

## Conclusion

The present findings revealed a superior health-related physical fitness and well-being for 12–15-year-old Danish girls and boys active in organized sports in their leisure time. These findings are consistent with research findings from other age-groups and provide evidence about the association between participation in organized sport and superior physical and mental health status. Furthermore, the results suggest that participation in soccer is superior to other types of sports in relation to cardiorespiratory fitness level, whereas participation in multiple sports and team sports in general are associated with higher well-being compared to individual sports. Consistent with existing evidence, girls presented with higher BMI and fat percentage, and lower physical fitness and well-being than boys. High rates of drop-out from sports among adolescent girls call for a special focus on this group in order to maintain good physical health and well-being.

## Abbreviations

ANCOVA: ANalysis of COVAriance
B: Boys
BMI: body mass index
CI: Confidence interval
d: Cohen’s d
DBP: diastolic blood pressure
F: F-statistic
G: Girls
IS: Individual sports
MAP: mean arterial pressure
MS: multisport
MVPA: moderate-vigorous intensity physical activity
NOS: No organized sports
OSP: Organized sports participant
OTS: Other team sports
PA: Physical activity
PAR: Parental support and autonomy domain
PHY: Physical well-being domain
PWB: Psychological well-being domain
RHR: resting heart rate
SBP: systolic blood pressure
SC: Soccer
SCH: School environment domain
SD: Standard Deviation
SE: Standard Error
SOC: Social support and peers domain
WHO: World Health Organization
Yo-Yo IR1C: Yo-Yo intermittent recovery 1 – children’s test
